# The evolving role of the immune microenvironment of tumor draining lymph nodes in the development of biomarkers of non-small cell lung cancer

**DOI:** 10.3389/fonc.2026.1803196

**Published:** 2026-05-15

**Authors:** Zhan Hao Xi, Benjamin Zollinger, Yusuke Koga, Jason Weis, Joshua D. Campbell, Sarah A. Mazzilli, Kei Suzuki

**Affiliations:** 1 Department of Medicine, Section of Computational Biomedicine, Boston University Chobanian & Avedisian School of Medicine, Boston, MA, United States; 2 Division of Thoracic Surgery, Inova Schar Cancer Institute, Fairfax, VA, United States

**Keywords:** immunology, lung cancer, lymph node-immune microenvironment, multiomics, non-small cell lung cancer, single cell sequence (scRNA-seq), tumor draining lymph node (TDLN), tumor-immune microenvironment

## Abstract

Non-small cell lung cancer (NSCLC) remains the leading cause of cancer-related mortality, and a substantial fraction of patients with resected early-stage disease experience recurrence despite curative-intent surgery. Current pathologic staging does not completely capture the biological heterogeneity that supports metastasis and drives relapse in node-negative patients. Development of robust prognostic and predictive biomarkers are needed to predict which early-stage patients are likely to progress and require additional treatment. Increasing evidence indicates that antitumor immunity is a major determinant of clinical outcome and therapeutic responsiveness. This is particularly relevant in the era of neoadjuvant, adjuvant, and perioperative immune checkpoint blockade where harnessing the potential antitumor properties of the immune system is essential. While most biomarker efforts have focused on the primary tumor alone, antitumor immune responses are orchestrated across multiple compartments, including tumor-surrounding lymph nodes, where antigen presentation, germinal center reactions including T and B cell priming and memory formation occur contributing to immunologic remodeling that can precede overt metastasis. Here, we review the cellular, transcriptional, and spatial architecture of the tumor–immune microenvironment (TIME) and lymph node immune microenvironment (LIME) in human NSCLC, emphasizing how immune cell composition, cell state, clonal dynamics, and spatial organization influence progression, recurrence risk, and response to immunomodulatory therapies. This review highlights the current technical and translational advantages and limitations of multimodal single cell technologies and discuss potential directions for early-stage NSCLC staging and optimizing therapy timing through leveraging TIME–LIME assessment utilizing multimodal technologies.

## Introduction

Lung cancer remains the number one cause of cancer related mortality in the United States (1–3). Patients with stage I Non-small cell lung cancer (NSCLC), primarily undergo resection of the tumor-containing lung in addition to regional and mediastinal lymph nodes (4–6). Despite receiving curative intent treatment during an early stage, nearly one in five patients with resected stage I NSCLC will have recurrence within five years (7). One of the limitations of the current pathologic TNM (tumor, node, metastasis) staging system is the lack of ability to accurately predict recurrence and clinical outcome in stage I patients. Identification of other indicators of aggressiveness in lymph node pathology beyond the presence of nodal and metastatic disease may be needed to improve the current TNM staging system (8, 9). Recent studies have worked toward identifying biomarkers of cancer aggressiveness focused on tumor surrounding lymph nodes (TSLNs) and have demonstrated the importance of host immune response and its relevance to prognosis (10). Analysis of the specific subtypes of tumor-infiltrating immune cells, such as exhausted CD8+ T cell infiltration, and certain immune markers expressed by the tumor cells, such as PD-L1, yields important prognostic relevance (11, 12). This work highlights the potential for investigating the host innate and adaptive immune response to the malignancy to define the host-tumor interaction in the TSLNs prior to metastasis to serve as a marker for tumor aggression and potential relapse (13–16).

The early diagnosis of early-stage NSCLC and assessment of the host immune response has shown promise for therapeutic intervention, however most pathologic assessments are focused on the immune microenvironment within the primary tumor alone (11, 17). This tumor-centric view only partially reflects the complexity of antitumor immunity, which is orchestrated across a network of compartments that includes the primary tumor and the TSLNs, where adaptive immunity matures (16). TSLNs serve as the primary sites of antigen presentation, T and B cell priming, and the generation of effector and memory responses, while also being the preferential sites for early metastatic colonization (18–22). Importantly, accumulating evidence suggests that lymph-node immune microenvironment (LIME) can undergo profound immunologic remodeling, such as development of immunosuppressive niches and disruption of lymphoid architecture prior to overt metastasis (23, 24). Such changes may influence the evolvement of the tumor immune microenvironment (TIME), and contribute to progression, and even recurrence, in patients staged as node negative.

Therefore, assessment of the TIME and LIME, which encompass both the primary tumor and TSLN, offer a more integrated framework for understanding disease biology and for developing novel prognostic and predictive biomarkers in NSCLC. Within this framework, the density of immune cell types, their activation states, interactions, and broader spatial architectures define the balance between pro- and anti-tumor processes. Recent advances in multimodal technologies such as single-cell RNA sequencing (scRNA-seq) and cellular indexing of transcriptomes and epitopes by sequencing (CITE-seq) offer the ability to deeply phenotype immune cell states at single cell resolution within TIME and LIME. Spatial platforms for spatial proteomics and spatial transcriptomics such as Standard BioTools Imaging Mass Cytometry and 10x Genomics Visium technology, can be used to understand the cell compositions underlying histological abnormalities and uncover novel cell-cell interactions or cell niches associated with aggressive phenotypes. Applied to paired tumor and lymph node specimens, these tools can identify immune cell types, cell states, and their spatial relationships, offering views of immune spatial niches that can become prognostic signatures associated with progression, recurrence risk, or response to immunomodulatory therapies. In this review, we summarize current knowledge on the TIME and LIME in NSCLC, using multimodal and spatial technologies, with a particular focus on its potential as a prognostic and predictive biomarker.

## Tumor and lymph node immune microenvironments

### Tumor immune microenvironment

The tumor immune microenvironment (TIME) describes the complex biological landscape of immune cells and tissue structures within and surrounding a tumor. The TIME is more than simply a geospatial layout of the cells, but instead it is comprised of evolving, dynamic interactions between the tumor cells, the immune cells, and the surrounding parenchyma as cell-cell signaling constantly changes downstream effects. Contributing to the TIME include various immune cell type compositions with different cell states, their spatial organization relatively to the tumor, and most importantly, their interactions with tumor and stromal cells such as cancer-associated fibroblasts, endothelial cells, and extracellular matrix components (23, 25–28). The TIME includes cell populations that are categorized as innate and adaptive branches of the immune system. Innate immune cells include natural killer (NK) cells, conventional and plasmacytoid dendritic cells (DCs), macrophages, neutrophils, myeloid-derived suppressor cells (MDSCs), and more recently identified, innate lymphoid cells (ILCs) (13, 29–31). The adaptive immune system is composed of CD4^+^ T cells, CD8^+^ T cells, and B cells, which require the innate immune system to be properly trained in the lymph nodes (32–34). The functional and phenotypic states of the innate and adaptive immune system have been extensively described in cancer and disease. These can include naïve, activated, effector, memory, exhausted, regulatory, and tolerogenic cell states which govern the role each subset contributed to tumor biology (35–40). Functionally, immune cells in the TIME tend to fall along a continuum between tumor-regressing (anti-tumor) and tumor-promoting (pro-tumor) roles (41–47). The immune cell state is often inferred from cytokine and chemokine secretion profiles (e.g., interferon gamma (IFN-γ) and tumor necrosis factor alpha (TNF-α) expression infer that CD8^+^ T cells are a cytotoxic lymphocytes, versus IL-10 and TGF-β inhibitory cytokines that support the function of T regulatory or suppressive cells) (26, 40, 47). The expression of inhibitory or activating receptors and ligands (such as PD-1, PD-L1, CTLA-4, TIM-3, CD80/CD86, OX40, and 4-1BB), can also be used to infer intrinsic effector cytotoxic potential, such as granzyme/perforin-mediated killing or antigen-presentation capacity (32, 41, 48–54). Importantly, these functions are not static: tumor evolution, metabolic constraints, chronic antigen exposure, and stromal remodeling can dynamically reprogram immune populations, shifting them from anti-tumor to pro-tumor phenotypes or vice versa, displaying great cellular transition plasticity (55–59). Defining the functional and phenotypic states and their spatial localization expands the descriptive view of “immune infiltration” to a functional concept of TIME. Thus, a robust characterization of TIME integrates cell type, state, spatial location, and temporal context, rather than relying solely on bulk composition. Therefore, we propose that these key attributes should be contemplated when discussing the TIME. First, cell identity, a higher level/single surface protein or gene alone is insufficient to define a cell, for example, CD8^+^ T cells can range from highly cytotoxic effectors to terminally exhausted cells with limited proliferative potential and impaired effector function (60–64). Second, spatial context matters: the same cell type can have distinct roles depending on whether it is localized within tumor nests, at the tumor–stroma interface, within tertiary lymphoid structures, or in perivascular regions (12, 65–70). Third, temporal dynamics and plasticity are critical: the TIME evolves with tumor growth, therapy exposure, and systemic immune status (28, 71–75). Therefore, to understand the clinical effects of the TIME on an anti-cancer directed immune response, one must parse the TIME into various individual parts. Each cell subtype and its cytokine expression can alter the immune response by either interacting directly with the tumor or by influencing a native partner cell. Some of the component parts of the TIME and their prognostic implications are discussed below.

### Lymph node immune microenvironment

Although most clinical and research efforts have focused on the TIME within the primary tumor, antitumor immunity is inherently distributed across multiple anatomical compartments and lymphoid structures that develop within the TIME (76). In NSCLC, the tumor immune landscape, which includes the lymph node (LN) immune microenvironment (LIME) and tertiary lymphoid structures (TLS), influences tumor progression (77, 78). The primary tumor provides the antigenic source and local context for immune recognition (79). Tumor cells release antigens through cell death or active secretion. Professional antigen-presenting cells (APCs), such as dendritic cells and macrophages, capture these antigens and process them for presentation on major histocompatibility complex (MHC) molecules (80). However, effective priming of naïve T cells typically occurs not in the tumor itself but in the LNs that drain the affected lung segments (81) and in the TLS. In the lymph nodes, APCs bearing tumor-derived antigens interact with naïve or memory adaptive immune cells, such as T cells in the paracortex, and B cells in the cortex region of lymph nodes (82). These interactions determine whether tumor antigens elicit productive effector and memory responses, are ignored, or induce tolerance (83, 84).The geospatial layout of the immune cells within the tumor-draining LN’s (TDLNs) and the relative cascade between APC’s, T cells, and B cells comprise the LIME. The LIME functions in a similar pattern to the TIME though it is remote to the tumor itself, at least initially. As seen in the TIME, reciprocal signaling can direct the immune response where pro- or anti-tumor cells are trained and activated. Once primed, effector adaptive populations recirculate to the tumor, where they confront additional layers of regulation, such as local checkpoint ligand expression, metabolic constraints (hypoxia, nutrient competition), suppressive myeloid populations, and physical barriers in the stroma (11, 85, 86). B cells and their progeny, including plasma cells, may provide tumor-specific antibodies, promote antigen presentation, or even illicit immune suppression (42, 87). Although the initial immune response from the TDLNs is kickstarted by APC’s, once malignant cells metastasize to the LN, these tumor cells will in turn insert themselves into the chemical signaling and drastically change the immune landscape in the LIME (88).

The TLS, like LN, acts as a local adaptive immune training hub to support local antigen presentation, affinity maturation of tumor-specific antibodies, and the maintenance of both CD4^+^ and CD8^+^ effector T cell populations within the tumor microenvironment (89–91). They are found in the surrounding parenchyma around the tumor instead of in draining LN basins. They are less mature than LN B cell follicles resulting in plasma cell dominated TLS or disorganized B cell aggregates that may be associated with immune suppression, altered antibody isotypes, or chronic inflammatory signaling that may facilitate tumor progression (92, 93). Furthermore, TLS’s cellular heterogeneity, and maturation state defined by presence of germinal center markers, follicular dendritic cells, and T follicular helper cells, all determine if the TLS will be tumor suppressive or progressive (90).

Furthermore, LIME shapes tumor TLS formation primarily by determining whether the TSLN preserves productive B cell activation, germinal-center output, and lymphocyte trafficking programs that can be relayed back to the tumor (94, 95). In NSCLC, metastasis in TDLN was associated with impaired maturation of intratumoral TLSs evident by fewer GC forming TLSs. Furthermore, CD19^+^ CD27^+^ memory cells shifted toward naïve B cell states in both TDLNs and tumors (94). Another study showed that mature TLSs in primary lung adenocarcinoma were associated with higher numbers of granzyme B positive cytotoxic lymphocytes in TDLNs, lower rates of nodal metastasis, and better clinical outcomes (95). These results suggest that favorable LIME promotes TLS formation and maturation through preservation of nodal GC competence and memory B cell formation. In contrast, LIME dysregulation is suggested to blunt these processes and impair TLS maturation. However, more studies directly analyzing tumor’s TLS and LIME in paired, spatially resolved fashion within the same patients will be needed to demonstrate this in order to resolve the link TLS and LIME biology to patient outcomes.

Immune interaction between the LIME and TIME is bidirectional. Feedback from the tumor microenvironment to LNs can in turn shape the phenotype of APCs and other immune cells that travel to the lymph nodes, leading to an iterative loop of bidirectional communication between tumor and lymph nodes (96–98). Regional lymph nodes also represent the preferential sites for early metastatic colonization (84, 98–100). Immunologic changes in these nodes can precede metastasis, such as remodeling of B cell follicles, accumulation of regulatory T cells, emergence of suppressive dendritic or myeloid populations, and alterations in stromal cell programs that are suggestive to establish a pre-metastatic niche that both facilitates future tumor spread and dampens systemic antitumor immunity (24, 80, 86, 99, 101).

Therefore, we propose that the TIME cannot be fully understood, nor can robust biomarkers be developed, by examining the tumor in isolation. A patient with a seemingly “inflamed” tumor may still have poor outcomes if regional lymph nodes harbor profoundly immunosuppressive niches that limit the generation of effective effector and memory responses (10, 67, 99). Conversely, favorable nodal immune architecture and robust germinal center activity might partially compensate for a less inflamed primary tumor by sustaining systemic immune surveillance (20, 102, 103). Furthermore, therapies that target only the tumor site (e.g., local radiation or surgery) may fail to address disease-relevant immune dysfunction residing in regional lymph nodes, and may require immune therapy for better patient outcomes (34, 104–107). Lastly, because both tumor and nodal tissues are often resected in early-stage NSCLC, this setting offers a unique opportunity to capture paired specimens and develop combined tumor-LN TIME biomarkers for prognosis and treatment selection.

## Immune cell states of the tumor and lymph node immune microenvironment of NSCLC

### T cell immunology in TIME

Early studies demonstrating the importance of immune infiltration on NSCLC survival and ultimately therapeutic response, were first established through conventional IHC studies that showed that higher densities of tumor-infiltrating CD8^+^ T cells are associated with improved survival (108–114). A widely used framework divides tumors into inflamed (immune hot), immune excluded, and immune desert (immune cold) categories (115–117). Inflamed tumors contain abundant immune cells in direct contact with malignant cells and often display strong inflammatory cytokines such as IFN-γ, that are sometimes accompanied by high expression of immune checkpoints, such as PD-1/PD-L1 (118, 119). Immune excluded tumors restrict CD8^+^ T cells to the surrounding stroma or the invasive margin, suggesting that stromal architecture, chemokine gradients, or vascular disruption restrict T cell infiltration into tumor nests (41). Immune desert show little CD8^+^ T cell presence in either tumor or stroma and often reflect limited chemokine recruitment or broader systemic immune dysfunction (41, 120–122). Thus, patients with inflamed tumors have greater survival and responsiveness to immune checkpoint blockade, while patients with tumors that are immune excluded or characterized as immune desert tumors, more often develop resistance to therapeutic intervention and/or experience early recurrence (17, 123–126).

More recent studies utilizing single cell and spatial profiling have revealed numerous CD8^+^ T cell states. T cell function and anti-tumor response depends less on total numbers and more on the distribution of distinct cell states and their spatial localization within the TIME or LIME. In addition to the neighboring cell types, all have prognostic value for TIME (127–141). In NSCLC, effector-like cells express cytotoxic mediators such as granzymes and perforins and inflammatory cytokines such as IFN-γ and TNF-α that support direct tumor killing (52, 127). Tissue resident memory like cells can express CD103 and CD69 and remain in epithelial niches where they can provide durable surveillance (142). Terminally exhausted or dysfunctional cells express multiple inhibitory receptors such as PD-1, TIM-3, LAG-3, TIGIT, and CTLA-4, as well as reduced expression of cytotoxic mediators such as granzyme or perforins and reduced inflammatory programs such as IFN-γ and TNF-α (63, 127, 143, 144). In contrast, progenitor exhausted cells marked by expression of TCF1 and PD1 retain proliferative capacity and can respond to immune checkpoint blockade (62, 63, 132, 145, 146). Spatial profiling suggests that these progenitor exhausted and tissue resident memory like populations are often positioned near antigen presenting cells in structures such as tertiary lymphoid aggregates or perivascular regions (147–150). (**Table 1**) Collectively, these findings underscore that where specific immune cells reside within the tumor, and which cellular neighborhoods they occupy, can be determinative of anti-tumor immunity and response to immunotherapy, thereby shaping patient prognosis and clinical outcome.

**Table 1:** Overview of adaptive immune cell types active in modulating immune response in the tumor-immune microenvironment.

Adaptive immune cells active in the tumor-immune microenvironment				
Cell type	Cell state/subset	Markers	Function (ref.)	Spatial localization
CD8+ T cells	Effector-like	GZMB PRF IFN-γ TNF-α	Direct tumor killing (127, 135)	Infiltration of tumor or restriction to stroma dependent on cytokine gradient
	Resident memory	CD103 CD69	Surveillance for local tissue protection (142)	Near antigen presenting cells within tumor
	Terminally exhausted	PD-1 TIM-3 LAG-3, TIGIT CTLA-4	Non-proliferative, decreased effector cytokine production & expression of inhibitory receptors (144)	Presence in both tumor infiltration and lymphoid tissue
	Progenitor exhausted	TCF1 PD1	Retain proliferative ability and increased PD-1 expression (62, 144)	Near antigen presenting cells within tumor or tertiary lymphoid structure (TLS)
CD4+ T cells	Th1 oriented	T-bet IFN-γ	Promote CD8+ cytotoxic activity & DC activation (33)	Secondary lymphoid tissue
	Th2 oriented	GATA-3, IL-4 IL-5, IL13	IgE upregulation, attraction of mast cells, eosinophils, and basophils (33)	Secondary lymphoid tissue
	Th17 oriented	STAT3 IL-17	upregulate anti-tumor IFN-γ, pro- tumor Tregs & infiltration of neutrophils (33, 152, 153, 157)	Secondary lymphoid tissue
Regulatory T cells (Treg)	Proliferative Treg	FOXP3 MKI67	Stimulate Treg population proliferation (158)	Co-localization with dendritic cells in tumor infiltration
	Effector Treg	FOXP3 PD-1, TIGIT CTLA4, LAG3	Suppression of anti-tumor response (162)	Co-localization with dendritic cells in TLS and stroma
	Chemokine specialized Treg	FOXP3 CCL17 CCL22	Induction of Treg recruitment & chemotaxis (154, 159)	Tumor margins
B Cell	Regulatory B Cell	IL-10	Suppression of immune response (170–172)	Tumor stroma; TLS
	Memory B Cell	CD27	Class-switched to recognize antigens (87, 171)	Increased frequency with proximity to tumor
Plasma Cell	Effector plasma cell	CD19^lo^ CD38^hi^	Immunoglobulin production (171)	Tumor infiltration
	Regulatory Plasma cell	CD138, PDL1 IL-10	Immune suppression (171)	Localization to tumor stroma over tumor nests

CD4^+^ T cells can differentiate into different cell states supporting tumor progression or regression (151). Th1 oriented cells promote cytotoxic immunity and support dendritic cell activation supporting and anti-tumor response, while Th2 and Th17 oriented populations can adopt context dependent roles that may encourage tumor progression (33, 152). Regulatory T cells (Tregs) defined by FOXP3 expression consistently contribute to local immunosuppression (153). Multiple Treg cell subtypes have been described in NSCLC that are transcriptionally and functionally distinct. These include proliferative Tregs with high MKI67 expression, effector Tregs with upregulated checkpoint inhibitors such as PD-1, TIGIT, CTLA4, and LAG3, and chemokine specialized Tregs that upregulate chemokines secretions such as CCL17 and CCL22 (154–159). Tregs can also be enriched at invasive margins, surrounding TLS, or near dendritic cell subtypes, forming immunosuppressive niches (160–162). An overview of active T cell types and cell states is summarized in **Table 1**.

Retrospective review of T cell subsets in resected tumors has highlighted that increased CD8^+^ tumor infiltrating cells and a high level of stromal CD8^+^ T cells have been suggested to be predictors of progression free survival (PFS) and overall survival (OS) (163, 164). Similarly, higher CD8^+^/CD4^+^ T cell ratios within tumor biopsies were associated with higher rates of pathological response in both metastatic NSCLC (165). Conversely, in a large cohort of 956 stage I adenocarcinoma patients’ high density of stromal FoxP3^+^ cells was associated with a higher risk of recurrence, though those with high stromal FoxP3^+^ and high CD3^+^ T cell infiltration had a similar recurrence rate to those with low stromal FoxP3^+^ T cells (166). Although simple ratios of CD8^+^ and CD4^+^ T cell subtypes or Treg density offer prognostic value, recent multimodal profiling indicates that more granular metrics integrating T cell states, including progenitor-exhausted and terminally exhausted subsets, their spatial organization relative to tumor cells and tertiary lymphoid structures, and their presence in TSLN’s LIME are valuable information for clinical actions and disease progression (167–169)(**Table 2**).

**Table 2:** Summary of key findings of the tumor-immune microenvironment (TIME) or the lymph node microenvironment (LIME) prognostic studies in non-small cell lung cancer (NSCLC) patients.

Authors	Year	Inclusion criteria	Study size	Key findings
Hu-Lieskovan et al. (163)	2019	Advanced NSCLC treated with pembrolizumab	N = 38	Tumor baseline PD-L1 expression and CD8+ infiltration were associated with improved PFS or OS, but not CD4
Hashemi et al. (164)	2021	Advanced NSCLC treated with pembrolizumab or nivolumab	N=141	Increased stromal and intra-tumoral CD8+ tumor infiltrating lymphocytes (TILs) were associated with improved PFS and OS, particularly with stromal TILs
Uryvaev et al. (165)	2018	Metastatic NSCLC or melanoma	N= 26 patients with NSCLC	A CD8+/CD4+ ratio > 2 and a CD8+ lymphocyte count > 886/mm^2^ was associated with higher response rates to anti-PD-1 therapy
Suzuki et al. (166)	2013	Resected stage I lung adenocarcinoma (LUAD)	N= 956	Increased recurrence risk with high density of FoxP3 expression. High tumoral IL-12Rβ2 was associated with improved RFS and high IL-7R was associated recurrence and worse OS
Thommen et al. (167)	2018	Stage IV NSCLC	N=21	The presence of CD8+ cells with high PD-1 expression had improved response to anti-PD-1 therapy and was associated with increased OS
Pelletier et al. (173)	2001	Resected NSCLC	N= 113	CD20+ B cell presence in peritumoral tissue was associated with improved OS, excluding squamous cell carcinoma (LUSC)
Liu et al. (174)	2021	Transcriptome profiles present in the The Cancer Genome Atlas for LUAD or LUSC	N= 512 LUAD; 497 LUSC	B cell infiltration was associated with improved OS in LUAD but not LUSC, and higher DC1 infiltration was associated with worse OS in LUSC
Backman et al. (175)	2021	Resected NSCLC	N= 357	Infiltration of natural killer and plasma cells were associated with longer OS in the whole cohort and LUAD patients but not LUSC. PD-L1 expression in LUSC was associated with longer OS
Dieu-Nosjean et al. (193)	2008	Early-stage NSCLC	N= 74	Increased density of intratumoral mature dendritic cells that cluster with T cells was associated with improved OS and DFS.
Kadota et al. (203)	2015	Resected solitary LUSC tumors	Training set (n =331) Validation set (n= 154	High tumor-infiltrating CD10+ neutrophil/low CD20+ lymphocyte ratio was associated with worse OS in both the training and validation cohort
Carus et al. (204)	2013	Resected stage I-IIIA NSCLC	N=335	Higher density of CD163+ macrophages in tumor or stroma was associated with increased nodal metastases but density of CD163+ or CD66b+ neutrophils were not correlated with RFS or OS
Pellinen et al. (205)	2023	Resected NSCLC	N= 350	Cancer associated fibroblasts (CAF)7 and CAF13 expression were associated with OS in stage IA-IB NSCLC
Cords et al. (206)	2024	NSCLC with at least 15 years follow up	N= 1070	Presence of tumor-like CAFs is associated with worse OS but inflammatory CAFs are associated with improved OS
Zhao et al. (207)	2023	Advanced NSCLC receiving PD-1 blockade therapy	N= 135	Higher fibroblast activation protein expression was associated with decreased CD8+ density, worse therapeutic response rate, and worse PFS, along with worse OS in LUSC
Reuben et al. (220)	2020	Treatment naïve NSCLC	N= 236	A closer intra-tumoral T cell repertoire homology with the surrounding uninvolved lung parenchyma was associated with lower OS
Reuben et al. (221)	2017	Localized LUAD	N= 45	Higher intra-tumoral T cell receptor heterogeneity was associated with lower DFS
Casarrubios et al. (223)	2021	NSCLC samples before and after neoadjuvant chemoimmunotherapy	N = 40	Decreased/uneven T cell receptor repertoire and a higher proportion of the top 1% most common T cell clones was associated with higher rate of complete pathological response, even more so than PD-L1 expression
Ruffini et al. (241)	2009	Resected NSCLC	N = 1290	The presence of TIL (almost entirely CD8+) was present in 23% of resected specimens and was associated with improved OS in LUSC
Hu et al. (101)	2022	Resected stage I NSCLC	N= 130	Lower CD3+ and CD8+ infiltration was associated with early recurrence within 3 years

OS, overall survival; PFS, progression-free survival; RFS, recurrence-free survival; DFS, disease free survival.

### B cell immunology in TIME

Studies have highlighted the co-modulation of B cells with T cells for clinical outcomes for NSCLC. Like T cells, diverse tumor-infiltrating B cells (TIBs) have been identified within the TIME. These span naïve, follicular, antigen-experienced memory states, diverse antibody-secreting plasma cells, and multiple suppressive regulatory B cells (Bregs) that can shape local T cell function and treatment outcomes (170, 171). Using single-cell profiling, research shows that with increasing proximity to tumor, naïve B cell frequency(CD19+ CD27-) decreases while memory B cells and plasma cell frequencies increase, with tumor-infiltrating plasma cells displaying more BCR repertoire diversity and less clonal dominance (87). Spatial analyzes in early-stage LUAD show that B cells are frequently concentrated into TLS through CXCL13 gradient, rather than being uniformly dispersed (171). TLS provides a structural scaffold that can enable local antigen presentation, affinity maturation, coordinated T cell and B cell interactions, and serve as hubs for B cell maturation and plasma cell differentiation inside the tumor (172).

Single-cell trajectory analysis indicates that plasma cells in the TIME are heterogeneous and exist on a functional spectra (171). In LUAD patients, early differentiating plasma cells (CD19^lo^, CD38^hi^, Ig-) may differentiate into effector plasma cells, or regulatory plasma cells (CD138^+^, PDL1^+^, IL10^+^), with effector plasma cell abundance having higher association with non-recurrence, and regulatory plasma cells and Bregs (CD20^+^, IL10^+^) present enriched in tumor nests (171)(**Table 1**). Breg subtypes are further differentiated based on expression of PD1, PDL1, CD5, Ki67, and CD38 (170). Therefore, as single-cell technologies unravel distinct intratumoral B cell subtypes, incorporating B cell subtype abundance into immune checkpoint inhibitors (ICI) treatment guidelines may improve patient outcomes (170).

In a cohort of 113 patients with resected tumors, Pelletier et al. found that across the entire cohort, general CD20^+^ B cell presence in the peritumoral tissue was associated with improved OS, though when grouped by non-squamous versus squamous lung tumors, the association was lost for squamous tumors (173). Similarly, B cell infiltration improved OS in adenocarcinoma patients but not in squamous cell patients (174). Backman et al. found improved OS with increased plasma cell infiltration in adenocarcinoma but not squamous cell carcinoma (175) (**Table 2**). Although, similarly to specific T cell lineages, B cells have a clear role in the clinical prognosis for NSCLC, the mechanisms are primarily through T cell modulation, however further insights into the interaction with the specific NSCLC histological subtype, may drive approaches for therapeutic intervention.

### Myeloid cell immunology in TIME

Myeloid cells include macrophages, dendritic cells, neutrophils, and myeloid-derived suppressor cells (MDSCs), and influence NSCLC TIME formation through influencing T and B cell priming, effector function, and tolerance (176, 177). Traditional M1/M2 macrophage dichotomies are now recognized as overly simplistic as a continuum of macrophage states with diverse roles in antigen presentation, phagocytosis, tissue remodeling, and immune regulation have been identified (178). Within NSCLC tumors, macrophages often segregate into antigen-presenting, inflammatory, and suppressive phenotypes (178, 179). Antigen-presenting macrophages express high levels of MHC-II and co-stimulatory molecules and may support local activation of tumor-reactive T cells (178, 179). Inflammatory macrophages are enriched for pro-inflammatory cytokines and chemokines such as TNF-α and CCL20, and can exhibit context-dependent effects that can either enhance tumor control or promote tumor progression (178). Suppressive macrophage states frequently express scavenger receptors, immunoregulatory ligands, and metabolic enzymes that attenuate T cell responses and foster tumor growth (178, 179). Among these, TREM2^+^ and SPP1^+^ macrophages consistently associate with immune suppression, stromal remodeling, and resistance to immunotherapy across cancer types, including NSCLC (180–184). Spatial imaging studies commonly localize these suppressive macrophages to tumor–stroma interfaces or in the vicinity of TLS, where they may impede effective T cell infiltration and TLS function (182, 185–187) (**Table 3**).

**Table 3:** Overview of innate immune cell types active in modulating immune response in the tumor-immune microenvironment.

Innate immune cells active in the tumor-immune microenvironment				
Cell type	Cell state/subset	Markers	Function (ref.)	Spatial localization
Macrophage	Antigen-presenting	MHC-II	Stimulate local T cells (178)	Tumor & stroma
	Inflammatory	C1QC FCN1	Pro-inflammatory (178)	Tumor & stoma
	Suppressive	CCL18 SPP1 TREM2	Pro-tumor: promotion of IL-10, angiogenesis, stromal remodeling, upregulation of tumor-associated fibroblasts (178, 180)	Tumor infiltration & TLS
Dendritic Cells (DC)	Conventional type 1 DC (cDC1)	CD141/BDCA1 CLEC9A	Antigen presentation via MHC-I for pro-cytotoxic processes (190)	Lymph nodes and non-lymphoid tissue
	Mature regulatory DC (mregDC)	LAMP3, MHC-II CD-86, PD-L1	Activates effector T cells (191, 193)	Co-clustering with CD8+ T cells, tumor infiltration
Neutrophils	Pro-tumor	VEGFA, MMP9 PD-L1	Angiogenesis, matrix remodeling, engage in tumor proliferation (194)	Can bind to tumor cells
	Anti-tumor	IFIT1	Responds to IFN-γ, and supports T cell response (194)	Direct interaction with tumor cells
Myeloid-derived suppressor cells	Monocytic	CD11b, CD14+	Immune suppression (199)	Tumor infiltration and circulation
	Granulocytic	CD11b, CD15 CD66 CD 14	Immune suppression, promote angiogenesis, escorting metastatic cells (199)	Tumor infiltration and circulation

Dendritic cells (DCs) form another critical axis of antitumor immunity through their capacity to prime and sustain tumor-specific T cell responses (188, 189). Conventional type 1 DCs (cDC1) are central to cross-presentation of tumor antigens and activation of cytotoxic CD8^+^ T cells (190). However, scRNA-seq has revealed mature regulatory DC (mregDC) defined by expression LAMP3 and MHC-II, co-stimulatory molecules such as CD86, and immunoregulatory molecules such as PD-L1 (191). These hybrid states can either support or suppress T cell responses depending on microenvironmental cues. The relative abundance of cDC1-like versus mregDC-like DCs in NSCLC tumors appears to influence the quality of intratumoral T cells and the degree of responsiveness to checkpoint blockade therapies (150, 191, 192) (**Table 3**). The clinical effects of mature DCs have also been shown; by staining for the protein DC-Lamp, which is expressed only by mature DCs that form clusters with T cells, Dieu-Nosjean and colleagues showed an association of density of intratumoral DCs with improved OS and DFS. Furthermore, they found higher overall density of T and B cells within DC-Lamp-high tumors (193) (**Table 2**).

Neutrophils and MDSCs further diversify the myeloid landscape. Neutrophils that are pro-tumor express VEGFA, MMP9, and PD-L1, that can promote angiogenesis, matrix remodeling, and T cell suppression (194, 195). Anti-tumor neutrophils express IFN stimulated genes and inflammatory cytokines (194–198). MDSCs, including both monocytic and granulocytic subsets, are frequently enriched in NSCLC and correlate with poor clinical outcomes and diminished responses to immunotherapy (199, 200) (**Table 3**). Single-cell and spatial analyzes of tumors and TSLN help discriminate suppressive MDSC populations from other myeloid lineages and have characterized their location to discrete immune niches such as vascular regions and Cancer-associated fibroblasts (CAFs) to promote metastasis (201, 202).

Different TIME myeloid cell lines have been linked with prognosis in NSCLC, but findings have been mixed. Using resected stage I-III squamous cell lung cancer tumors (training cohort n = 331, validation cohort n = 154), Kadota et al. found that a high CD10+ neutrophil/low CD20+ lymphocyte ratio in the TIME was associated with worse OS in both the training and validation cohort (hazard ratio [HR] 1.61, p = 0.006; HR 1.75, p = 0.043, respectively) (203). Meanwhile, in 335 resected stage I-IIIA NSCLC, the densities of CD66b + neutrophils and CD163+ macrophages were not associated with recurrence free survival (RFS) or OS (204) (**Table 2**).

## Stromal alteration and lymph node architecture that influence the TIME and LIME

### Stromal alterations that contribute to TIME and LIME

Other than immune cells, tumor suppressive and progressive fibroblasts and endothelial cells also contribute to the immune landscape in the TIME and LIME. CAFs shape chemokine gradients, contribute to extracellular matrix deposition that restricts T cell entry, and secrete factors that influence recruitment and polarization of myeloid cells (201). There is a large heterogeneity in CAF subtypes and their phenotypic presentation. However, multiple different CAF subsets have been associated both positively and negatively with prognosis in NSCLC (205, 206). As an example, NSCLC patients received anti-PD-1 blockade with a high expression of fibroblast activation protein (FAP), a marker of active CAFs, had a shortened median PFS (6 vs. 22 months, p< 0.0001) (207).

Alterations in the vasculature of a tumor through differential expression of adhesion molecules and checkpoint ligands, governs lymphocyte trafficking, retention and contribute to invasion and metastasis (208, 209). Stromal cells of the LN include fibroblastic reticular cells (FRCs), high endothelial venules (HEVs), and lymphatic endothelial cells (LECs), which shape antitumor immunity through their changes in cell state, morphology, and localization and contribution to LN architecture (23, 210, 211). They are among the earliest responders to tumor-derived signals (23). FRCs, the dominant fibroblast population in LN’s T cell zone, maintain the conduit network and produce the chemokine and cytokine cues that orchestrate T cell–DC interactions (23, 210). In TDLNs, FRCs remodel to myofibroblast-like transcriptional profile, even before metastatic spread, characterized by increased extracellular matrix deposition, altered conduit and B cell follicular architecture, and skewed chemokine production (24, 212). Changes include reduced CCL19 and CCL21 and increased CXCL12 and TGF-β that disrupt T cell motility, and misguide DC localization, dampening efficient priming of tumor-reactive T cells, and B cell follicular health (213). These CCL19 expressing FRCs have been shown to influence T cell localization, activation, survival, and sustaining effector function within the NSCLC TIME specifically (214). FRC may also express PD-L1, promoting CD8+ T cell exhaustion, contributing to formation of pre-metastatic niche in lymph nodes (215). HEVs lose their specialized morphology and downregulate peripheral node addressin (PNAd) and CCL21, impairing naïve lymphocyte recruitment and infiltration, while LECs upregulate PD-L1 and antigen scavenging programs that reinforce peripheral tolerance (216–218). Together, these transcriptional and spatial alterations in the stroma create an immunosuppressed, architecturally distorted, premetastatic niche of LIME that limits productive antitumor responses and influences how immunity in LIME influences TIME. Overall, human NSCLC tumors contain highly complex and spatially organized immune ecosystems in which T cell, B cell and TLS, myeloid, and stromal networks interact to either promote or restrain disease progression.

### Alterations in the lymph node architecture that influence the TIME & LIME

TSLNs are central hubs where innate immunity trains the adaptive immunity, with the LN stroma orchestrating the interaction. Therefore, the LIME of TSLN is essential to control tumor progression and should not be considered prognostically as merely metastatic grounds for cancer. Because they are routinely resected together with the primary tumor in early-stage NSCLC, TSLNs represent a uniquely accessible and underutilized biomarker space. TSLNs frequently exhibit structural and cellular changes that reflect chronic antigen exposure, inflammation, and tumor-induced immune modulation. These changes can be observed in both non-metastatic and metastatic nodes and may precede overt tumor cell colonization. Non-metastatic nodes often show reactive hyperplasia with secondary B cell follicles evident by prominent germinal centers (GC), consistent with ongoing immune responses to tumor and other infections, but closer examination reveals altered GCs. Some LN B cell follicles display decorticated B cell follicles, and enriched plasma cell presence suggesting dysregulated humoral responses (24). T cell zones may be enriched for Tregs, exhausted or anergic T cell subsets, and dendritic cell states that promote tolerance rather than effective priming. In metastatic nodes, metastatic tumor cells disrupt normal LN architecture, leading to collapse of B cell follicles, reorganization of T cell zones, and accumulation of suppressive myeloid cells, and distortion of normal stromal structure. Spectrum of metastatic nodes ranges from preserved lymphoid structure with active immunity to complete immune depletion. In pre-metastatic and metastatic niches, tumor secreted cytokines, chemokines, exosomes, and metabolites reach TSLNs before tumor cells. These can induce expansion of Tregs and MDSCs, reprogram DCs and macrophages toward tolerogenic phenotypes, remodel FRCs, and alter B cell follicle dynamics. This remodeling inhibits generation of effective effector and memory T cells, skew B cell responses, and creates a permissive environment for subsequent tumor cell seeding. Crucially, these changes can occur in histologically negative nodes, implying that TSLNs may harbor early, biologically meaningful alterations that are invisible to current standard pathologic staging but relevant to progression and recurrence risk in node-negative patients due to LIME alterations. Highlighting the importance of histologically negative nodes and the ability to continue to produce functional lymphatic architecture free from tumoral interference, a study by He and colleagues with a cohort of 616 patients with resected NSCLC tumors and associated systemic lymph node dissection or sampling, found an association with pathologically negative nodes in resected specimens and mature intra-tumoral TLS. In turn, more mature TLS was associated with improved survival (94). This study underlines the important relationship and bidirectional signaling between the LIME and TIME. A LIME free from signaling disruption from metastatic cells aids in the development of a mature and active TIME.

## Significance of tumor heterogeneity on the tumor immune microenvironment in NSCLC

Beyond the value of assessing individual cell types, overall TIME T cell diversity to recognize antigens has also been implicated as a marker for responsiveness to treatment. Higher intratumoral T cell receptor clonality or recognition and expansion toward a specific was associated with a reduced percent residual tumor at surgical resection and increased rate of major pathologic response after neoadjuvant ICI (219). Similarly, a closer homology between the intra-tumoral T cell and uninvolved lung parenchymal T cell repertoire was associated with lower OS. Furthermore, greater T cell clonality was found with higher counts of CD8+ T cells and lower clonality with *EGFR* mutated tumors (220). The same group also found a higher level of intra-tumoral T cell receptor heterogeneity was associated with lower RFS (221). Together, these findings suggest that a higher and more concentrated T cell clonal expansion indicate that a more tumor-focused response could be launched in case of recurrence (220, 222). In a similar vein, using samples from the NADIM trial for neoadjuvant immunotherapy, Casarrubios and colleagues investigated T cell receptor repertoire and pathologic response (223). They found that patients with complete pathologic response had less T cell receptor evenness in the repertoire and an increased proportion of the top 1% most common T cell clones. They also found a model using the percent of the top 1% clonal space was more predictive for complete pathologic response than PD-L1 expression or tumor mutational burden (area under the curve of a receiver operating characteristic curve (AUC) = 0.967, 0.767, 0.550 respectively) (223). These studies highlight the impact that the presence of targeted T cell clonotypes within the TIME can have on clinical outcomes (**Table 2**).

With the rise of immunotherapy as an integral part of treatment for both resected and non-resected NSCLC, the interplay between the tumor phenotype/genotype and TIME has gained a significant clinical focus. Several large multicenter randomized controlled trials (RCT’s) have established improved OS and event-free survival (EFS) benefits with the use of ICI for NSCLC. ICI’s trialed include atezolizumab, pembrolizumab, nivolumab, and durvalumab in the adjuvant, neoadjuvant, and perioperative settings (224–230). Although these studies have all shown clinical benefits with ICI across patient populations, questions remain about which subgroups of patients are the ones benefiting the most from these therapies. Therefore, an improved understanding of the TIME and the development of prognostic as well as predictive markers is vital for predicting an individual patient’s response to these new treatments. For example, two such biomarkers that have been shown are that patients with *EGFR* or *ALK* mutations respond poorly to ICI and as such, patients with those mutations were excluded from several of the aforementioned trials (231, 232). Beyond these two common genetic mutations, the identification of patients who may have a stronger or weaker response to treatment and a higher or lower risk of recurrence has become a very salient topic of investigation (224, 226, 227). It is known that patients can have a wide range of responses to ICI (227, 227, 231–235). Particularly, in the CheckMate-816 trial, which was a phase III trial investigating survival benefits of neoadjuvant nivolumab in resectable stage IB-IIIA NSCLC, only 24.0% and 36.9% of patients receiving neoadjuvant nivolumab + chemotherapy had a complete or major pathological responses. In patients who did not have a pathological complete response, EFS was not significantly different between those undergoing neoadjuvant chemoimmunotherapy or chemotherapy alone (227). Using single-cell and spatial technologies, identifying predictive biomarkers in the pretreatment setting could greatly improve patient care. Potential ways these could be integrated include by optimizing the timing and intensity of neoadjuvant/adjuvant therapy, reducing treatment-related toxicity, enabling escalation or de-escalation strategies, preserving eligibility for curative-intent interventions and clinical trials, and strengthening individualized prognostic counseling and follow-up planning.

The TIME has become a target for such an investigation for biomarkers for predicting responsiveness to treatment, prognosis, and possible new therapeutics. Protein expression on the tumor itself, local infiltrating immune cells, and local stromal cells have all been implicated in prognosis. One obvious subject for investigation is the PD-1/PD-L1 axis, as this is the target of the approved ICI. Currently, the exact relationship between the relative PD-L1 expression and the response to ICI is unclear. A meta-analysis of eight RCT’s found that all patients treated with neoadjuvant chemoimmunotherapy had improved EFS and pathologic complete response rates regardless of PD-L1 expression, even in those with ≤ 1% of tumor cells, but only those with PD-L1 ≥ 1% of tumor cells had an improvement in OS (236). However, those with 1-49% and ≥50% did not have significantly different EFS (236). When examining individual RCT’s though, there have been inconsistent findings with PD-L1 expression and outcome (236). The phase II NADIM trial (patients n = 46) and pilot neoadjuvant nivolumab trial (n= 21) showed no correlation between EFS and PD-L1 expression (237–239). However, the phase II NEOSTAR trial found fewer viable tumor cells within tumors with PD-L1 > 1%, and the larger phase III CheckMate-816 trial found improved EFS with PD-L1 > 1% (239, 240). Furthermore, when examining perioperative ICI, both the KEYNOTE-671and CheckMate-77T phase III trials found a relative improvement in EFS with increasing levels of PD-L1 expression (224, 225). Although PD-L1 expression is clearly a biomarker for responsiveness to ICI, there is no linear relationship between expression and definitive positive outcomes. Further understanding of the tumor-immune interaction must be parsed out. Beyond simply the PD-1/PD-L1 axis and the predicting responsiveness to ICI, other aspects of the TIME and protein expression by both immune and tumor cells has been strongly associated with general oncologic outcomes in both early and more advanced stage NSCLC. As a result of the aforementioned trials, ICI’s have become part of the standard of care for advanced stage NSCLC (stage IB and above) in appropriately selected patients. However, the TIME also provides important information for prognostication in early-stage cancer as well. In a review of 1290 patients, Ruffini et al. found an improvement in OS for stage I squamous cell carcinoma patients with the presence of tumor-infiltrating immune cells (241). Hu et al. found that a lower density of tumor cell infiltration was associated with early recurrence (within 3 years) in resected stage I patients (101). The TIME is strongly associated with overall prognosis, and we will highlight some of the individual components of the TIME and TSLN and how their presence can modulate clinical outcomes for patients (**Table 2**).

### Clinical prognostic potential of TIME and LIME

Both the TIME and LIME offer prognostic potential for patients with NSCLC based on their composition and relative cytokine expression. In the current era of ICI integration and changing oncologic resection patterns, researchers are working to identify biomarkers for both overall survival with the malignancy and to predict responsiveness to treatment. Most of the above studies used resected specimens, identified the presence of specific cell morphologies or signal expression, and then found association with survival. A summary table of the major above prognostic studies is provided in **Table 2**. A major future direction to clinically integrate TIME or LIME is the identification of immunological signals from the preoperative biopsy. Clinical management of NSCLC lends itself well to making use of immunological markers in both the TIME and LIME. Unless there are anatomical barriers, most lung lesions are usually able to be biopsied either by ultrasound or navigational-guided bronchoscopy or by computed tomography (CT) guidance. With bronchoscopy, the mediastinal nodes are typically staged and biopsied concurrently, or if any lymphadenopathy is noted on imaging workup, mediastinal staging is standard. Therefore, these biopsy tissues offer opportunity to use immunological biomarkers while still in the pre-treatment stage. Potential biomarkers could include information how potential T cell clonotypes potentiate responsiveness to ICI. For peripheral NSCLC > 2 cm in size, sub-lobar resections have been shown to be adequate (242, 243). However, if the biopsy can offer information about the aggressiveness of the tumor and possible downregulation of an immune response, clinicians may opt for an anatomic resection to increase lymph node harvest and ensure no cellular nodal metastases were missed (209). For locally advanced disease with nodal metastasis, an understanding of how the metastatic cells interact with the LIME can inform when progression to distal spread might be expected or tumor response to systemic therapy. There are many opportunities for prognostic integration of the TIME and LIME into clinical management of NSCLC, and as technology for analyzing smaller amounts of tissue continues to progress, the opportunities will grow.

## Technological frontiers in defining the TIME

The paradigm shift in non-small cell lung cancer (NSCLC) oncology from non-specific cytotoxic agents to immunotherapy has necessitated better profiling of TIME. Accurate characterization of TIME across both the primary tumor and TSLN of NSCLC requires tools that can resolve cell identity, cell state, and spatial context at single-cell resolution. Bulk transcriptomic assays provide an average of the immune landscape, often obscuring the critical cellular heterogeneity and spatial nuances that dictate therapeutic response. Other assays such as immunohistochemistry (IHC) can provide spatially resolved expression but only for a limited number of markers at a time. Recent advances in single-cell RNA sequencing (scRNA-seq), CITE-seq, and spatial genomics/proteomics enable multimodal profiling of tumors and TSLN, revealing immune cell states and spatial niches that are not accessible by conventional approaches (244, 245) (**Table 4**).

**Table 4:** Comparison of genomic, transcriptomic, proteomic, and multiomic approaches for investigating the tumor-immune microenvironment in non-small cell lung cancer (NSCLC).

Technology	Main data layers	Most applicable scenarios in NSCLC	Key strengths	Main limitations
Bulk genomics & transcriptomics	DNA mutations/copy number variations/fusions & RNA expression	Molecular subtyping; driver alteration discovery; tumor classification; treatment stratification in large cohorts; comparison of LUAD versus LUSC.	Mature pipelines; compatible with FFPE and large retrospective cohorts; relatively scalable; links genotype to transcriptional consequences.	Averages across mixed cell populations; weak resolution of rare cell states; cannot resolve cell–cell interactions or spatial architecture; vulnerable to tumor purity effects.
scRNA-Seq	mRNA sequencing with paired cell surface protein expression, TCR/BCR sequencing	Dissecting tumor heterogeneity; mapping immune cell states; identifying rare resistant clones; studying T cell exhaustion, B cell states, and myeloid diversity in TIME/LIME.	Resolves rare populations and transitional states; enables lineage/state inference; can connect phenotype with clonality or regulatory state.	Tissue dissociation biases; loss of spatial context; expensive and analytically intensive; small patient numbers are common; fresh tissue is often preferred to preserve delicate cell types.
Spatial Transcriptomics	High Resolution spatial RNA mapping	Building NSCLC tissue atlases; locating malignant, stromal, and immune programs; identifying spatially restricted niches; comparing tumor core, invasive margin, TLS-adjacent, and normal-adjacent regions in TIME. Identify abnormal LIME features, including follicular architecture, pre-exhausted immune subsets, myeloid suppressive niches, and differences between TSLN, distant LNs.	Combines enhanced resolution similar to scRNA-seq, with preserved tissue architecture for spatial profiling; especially strong for TIME/LIME studies.	Spatial transcriptomics often have lower resolution than true single-cell assays and may capture mixed spots; deconvolution depends on reference quality; cost and computational burden remain substantial.
Spatial Proteomics	High Resolution spatial protein mapping	Identifying cell membrane enhances cell boundary segmentation; mapping checkpoint-ligand geography; validation of immune niches; studying cellular neighborhoods, and clinically relevant cell–cell proximity patterns.	Directly visualizes cell-cell interactions, tissue architecture, and cell neighborhoods; strong for biomarker discovery tied to location rather than abundance alone.	Marker panels are constrained and hypothesis-driven; antibody validation and segmentation errors can affect results; throughput is lower than bulk assays; cross-study standardization remains challenging.
Spatial multi-omics	Spatial RNA + spatial protein	Mechanistic studies of tumor ecosystems; immunotherapy response modeling; analysis of coordinated tumor–stromal–immune organization; high-resolution biomarker discovery.	Most comprehensive view of “who is where and doing what”; well suited to uncovering spatial crosstalk and ecosystem-level predictors of outcome.	Highest cost and technical complexity; difficult data integration; limited availability of matched high-quality samples; often low cohort size, which can hinder validation and generalizability.

LUAD, lung adenocarcinoma; LUSC, lung squamous cell carcinoma; FFPE, formalin fixed paraffin embedded; TIME, tumor-immune microenvironment; LIME, lymph node-immune microenvironment; TLS, tertiary lymphoid structure; TSLN, tumor surrounding lymph nodes; LN, lymph node.

### Conventional multiplex staining and flow cytometry for TIME assessment

Single-marker IHC with conventional cell surface markers (e.g. CD3, CD8, FOXP3, PD-L1, etc.) has been used to quantify immune infiltration, estimate the balance between effector and regulatory T cells, and assess expression of immune checkpoint ligands on tumor and immune cells is currently the primary tool used by clinical pathologist (246, 247). In parallel, flow cytometry has historically served as a foundational immunology assay for defining and functionally annotating immune cell populations, enabling multiparameter quantification of lineage markers, activation/exhaustion phenotypes, and intracellular cytokines at single-cell resolution in dissociated tumor or lymph node specimens. Although flow cytometry provides richer phenotyping than single-marker IHC and underpins much of the canonical marker logic used in tissue studies, it typically requires fresh or viably preserved material, and is constrained in multiplexing depth by spectral overlap and fluorescence spillover.

Semi-quantitative scoring or digital image analysis can stratify tumors into broadly “immune-rich/hot” or “immune-poor/cold” categories and has shown prognostic relevance in several solid tumors, including NSCLC (74, 166, 248–250). Multiplex IHC and chromogenic or fluorescent panels expanded this concept by allowing simultaneous visualization of a limited number of markers in formalin-fixed, paraffin-embedded (FFPE) tissue (251, 252). RNA *in situ* hybridization approaches, such as RNAscope, further complement protein-based assays by enabling sensitive, spatially resolved detection of specific transcripts in FFPE sections, facilitating cell-type annotation and pathway readouts when high-quality antibodies are unavailable or when transcriptional states are of particular interest. Although these approaches preserve spatial information and can distinguish immune infiltrates in tumor nests versus stroma or invasive margins, they remain constrained by the number of targets that can be evaluated in parallel in routine workflows and are often focused on a selected set of antigens/transcripts rather than unbiased, high-dimensional profiling.

### Latest deep phenotyping tools for TIME/LIME assessment

Building on the tremendous impact of bulk RNA, DNA and TCR/BCR profiling has contributed to our refined understanding of the TIME/LIME, single-cell RNA sequencing (scRNA-seq) has enabled the capturing of the transcriptome of thousands of individual cells in an unbiased fashion and can reveal novel cell types or activation states within complex tissues. For example, scRNA-seq has been used to differentiate between pre-exhausted and terminally exhausted CD8+ T cells. While both subsets express PD-1 (*PDCD1*), the pre-exhausted cells express high *TCF7*, *LEF1* gene, and low *TOX*, in contrast to terminally exhausted cells which have opposite pattern of expression (253–257). For patients given ICI treatments such as pembrolizumab, nivolumab, and cemiplimab, the pre-exhausted CD8^+^ T cells can proliferate and give rise to effector CD8^+^ T cells, leading to better patient outcomes (258). Therefore, patients with higher pre-exhausted to exhausted CD8^+^ T cell ratios respond better to ICB therapy (259, 260). In the context of NSCLC, where intratumoral heterogeneity (ITH) is a hallmark of disease progression and drug resistance, scRNA-seq has moved the field beyond broad categorizations, such as lymphocyte enriched or depleted, to precise immunophenotyping.

While scRNA-seq offers depth, it suffers from “dropout” events where lowly expressed genes are missed, and mRNA levels do not always correlate with protein abundance due to post-transcriptional regulation (261)(**Table 4**). Cellular Indexing of Transcriptomes and Epitopes by Sequencing (CITE-seq) bridges this gap by using oligonucleotide-conjugated antibodies to simultaneously sequence cell surface proteins and the transcriptome from the same cell (262, 263). CITE-seq is particularly valuable for differentiating immune cells that are in transcriptionally similar but functionally distinct states (264). For example, distinguishing between certain memory T cell subsets or NK cell subpopulations can be challenging based on RNA alone due to the transient nature of some gene transcripts. CITE-seq provides the definitive surface protein expression, such as CD45RA, CD45RO, CD56, CD16 that are needed to annotate these clusters accurately. Similarly, important checkpoint molecules like LAG3 and CTLA4 often have low transcript counts even in cells where the protein is functional and present on the surface. Additionally, when CITE-Seq is combined with T cell receptor (TCR) and B cell receptor (BCR) sequencing, CITE-seq enables tracking of clonally related lymphocytes across tumor and TSLN. This makes it possible to map pathways of T cell priming and differentiation, such as from naïve or central memory clones in TSLN, through progenitor-exhausted states, to terminally exhausted effectors in the tumor, to understand how nodal immune architecture supports or constrains these trajectories. Additional emerging multiomic platforms integrate additional layers, such as chromatin accessibility (scATAC-seq), to link epigenetic landscapes to immune cell states, with potential implications for understanding durable versus transient reprogramming of TIME, and LIME.

CITE-seq and scRNA sequencing applied to regional lymph nodes have begun to resolve these changes at the level of cell identity and state. In the T cell compartment, single-cell analyzes typically identify naïve, central memory, effector, regulatory, and T follicular helper populations, along with progenitor-exhausted subsets (265). Comparisons between tumor and TSLNs reveal clonotype sharing between tumor-infiltrating CD8^+^ T cells and TSLN T cells, supporting the notion that TSLNs are the source of intratumoral effector and exhausted populations (266). TSLNs from patients with more advanced or aggressive disease often show enrichment of Tregs and pre-exhausted naive CD8^+^ T cells, even in the absence of metastasis, and display altered transcriptional programs related to stimulation, inhibition, cytotoxicity, metabolism, and migration (151, 267). In the B cell compartment, single-cell profiling distinguishes naïve, germinal center, memory, and plasma cell states (268). TSLNs may exhibit loss of classical GC architecture in various cancers, resulting in expansion of exhausted B cell phenotypes and disproportionate accumulation of plasma cells with restricted receptor repertoires, along with altered expression of antigen presentation molecules and co-stimulatory or co-inhibitory ligands that influence their role as antigen-presenting cells (24, 269–272).

Myeloid and stromal compartments in regional lymph nodes are similarly remodeled. Single-cell datasets identify mregDCs enriched in nodes from high-risk patients of various cancers, as well as monocyte and macrophage subsets expressing immunoregulatory molecules, scavenger receptors, and cytokines that influence T cell dysfunction and skew B cell responses (96, 273, 274). Changes in FRCs and related stromal subsets alter chemokine landscapes and influence lymphocyte positioning (275). Together, these findings support the idea that regional nodes can transition from an immunologically competent state that supports effective priming and memory formation to a reprogrammed, immunosuppressive state that undermines antitumor immunity. Many of these features are quantifiable and thus represent candidate biomarkers that could be incorporated into composite LIME and TIME prognostic signatures.

### Spatial transcriptomics and highly multiplex spatial proteomics to assess TIME/LIME

Although scRNA-seq and CITE-seq offer deep phenotyping of individual cells, the assays require the dissociation of cells which removes important spatial context. Spatial transcriptomics (ST) platforms, such as 10x Genomics Visium (55 um spots) and Visium HD (2um bins) using probe based and 3’ direct profiling of the whole transcriptome spatial analysis, or the Nanostring GeoMx Digital Spatial Profiler (DSP) (50–100 cells), or 10x Gemonics Xenuim (0.5-1um) that employ targeted gene panel approaches (**Table 5**), to map gene expression to histological coordinates (276, 277). In lung cancer, these technologies have revealed that the immune composition of the tumor core is distinct from the invasive margin and the adjacent normal tissue (278–284). For example, the employment of GeoMx DSP on tissues from patients treated with bispecific antibodies demonstrated that gene signatures derived from the stromal regions were more predictive of therapeutic response than those from the tumor core, revealing that the “barrier” functions of the stroma are as critical as the effector functions within the tumor nests (285–287). Moreover, ST can profile the maturation state of TLS, which are aggregates of T and B cells that serve as local factories for adaptive immunity, and distinguishing between immature aggregates and mature, germinal-center-containing TLSs that correlate strongly with survival (288–294). Recent multimodal spatial-omics revealed KRT8-expressing Alveolar Cells (KACs), also pathologically known as Reactive Type II Pneumocytes (RPII), as the earliest progenitor cells of LUAD that develops due to proinflammatory microenvironments caused by IL-1β secreting macrophages (295). Other examples include identifying vascular invasion (VI) signature for early-stage NSCLC, and gradients of interferon signaling that identify immune hot and cold tumor regions (209, 296, 297).

**Table 5:** Summary of spatial transcriptomic and spatial proteomic technologies.

	Technology		Target	Amenable tissue	Spatial resolution	Capture area	Gene coverage	Key advantages	Key limitations
Spatial Transcriptomics	Sequencing Based	*10x Genomics* Visium v2	RNA	FFPE/FF (pr) FR (pr/3’)	~55 µm spot (1–10 cells)	6.5 x 6.5 mm 11 x 11 mm	Whole transcriptome probe based/3’ Poly(A)	Widely adopted robust chemistry; integrates well with scRNA-seq deconvolution	Not single-cell; spot mixing; limited sensitivity for rare populations
		*NanoString* GeoMx DSP	RNA	FFPE, FR	ROI-based (10–600 µm)	0.66 x 0.79 mm	Targeted Panel up to ~1,800 genes Whole transcriptome	Works on FFPE; flexible ROI selection	No true single-cell resolution; ROI averaging
		*Curio* Seeker (Slide-SeqV2)	RNA	FR	~10 µm	3 x 3 mm 10 x10mm	Whole transcriptome	Higher resolution than Visium; unbiased transcriptome & no instrumentation	Lower capture efficiency, technically demanding, specialized slides need for tissue sectioning
		*10x Genomics* Visium HD	RNA	FFPE/FF (pr) FR(pr/3’)	~2 µm	6.5 x 6.5 mm	Whole transcriptome probe based/3’ Poly(A)	Near single-cell resolution; improved spatial fidelity	Computationally intensive; still emerging workflows
		*STOmics* Stereo-seq	RNA	FFPE, FR	~0.5–10 µm	10 x 10 mm 13 x 13 mm	Whole transcriptome probe based	Ultra-high resolution; large tissue coverage	Large data size; limited global adoption
	FISH Based	*Vizgen* MERFISH	RNA	FR	~0.5–1 µm	10 mm^2^ 30 mm^2^	100–10,000 genes	Subcellular localization; high sensitivity	Targeted gene panels; not whole transcriptome
		*Spatial Genomic* seqFISH+	RNA	FFPE, FR	~0.5–1 µm	ROI - based on imaging modality	Up to ~10,000 genes	Very high multiplexing; cellular context preserved	Expensive; complex probe design, more complex microscopy needs
	Imaging Based	CosMx SMI	RNA	FFPE, FR	~0.5–1 µm	15 x 20 mm	Targeted Panel up to ~6,000 genes Whole transcriptome	High sensitivity; FFPE compatible	Cost; targeted design
		*10x Genomics* Xenium (10x)	RNA	FFPE, FR	~0.5–1 µm	10.45 x 22.45 mm	Targeted Panel up to ~5,000 genes Custom up to 480 genes	High per-gene sensitivity: large capture area	Number of genes detected, specialized slides and high cost equipment needed
Spatial Proteomic	*Ionpath* MIBI-scope		Protein	FFPE	~0.5–1 µm	0.8 x 0.8 mm	up to ~40–50	High signal-to-noise; re-imaging possible	Specialized instrumentation
	*Akoya* PhenoCycler (CODEX)		Protein	FFPE, FR	~0.25-1 µm/pixel	35 x 18 mm	up to ~100	Large panels; iterative imaging; intact tissue	Antibody validation burden; imaging time
	*Standard BioTools* Imaging Mass Cytometry (IMC)		Protein	FFPE, FR	1 µm	ROI selection or whole tissue section	up to ~40–50	Single-cell resolution; minimal signal overlap	Tissue destruction; limited field size
	t-CyCIF		Protein	FFPE	~0.5–1 µm	ROI - based on imaging modality	up to ~100	Uses standard microscopes; scalable, no propriety methods of expensive kits	Long acquisition; photobleaching
	*Nanostring* GeoMx DSP		Protein	FFPE, FR	ROI-based	0.6 x 0.79 mm	up to ~100	clinically scalable	Not single-cell resolution
Spatial Multi-Omics	*AtlasXomics* Spatial-coProfiling		RNA + ATAC	FR	25 µm	5 x5 mm	Whole transcriptome/ATAC	Commercial ATAC spatial platform	Microfluidics complexity
	*10x Genomics* Xenium (10x)		RNA + Protein	FFPE, FR	~0.5–1 µm	10.45 x 22.45 mm	Targeted Panel & Custom genes + 27 Protein Immune/Cancer Panel	High per-gene sensitivity: large capture area	Targeted panel only
	*Nano String* CosMx SMI		RNA ± Protein	FFPE, FR	Single-cell	15 x 20 mm	Targeted or Whole transcriptome Protein Panels up to ~68	High sensitivity; FFPE compatible	Cost; targeted design
	*Standard BioTools* IMC		RNA + Protein	FFPE, FR	~1 µm	ROI or whole tissue section	Up to ~40–50 protein or mRNA single targets	Single-cell resolution; minimal signal overlap	Tissue destruction; limited field size

Key technical details, advantages, and limitations of selected spatial transcriptomic and proteomic platforms are presented. FFPE, formalin fixed paraffin embedded; FF, fixed frozen; FR, fresh frozen; Pr, probe.

**Figure 1:**
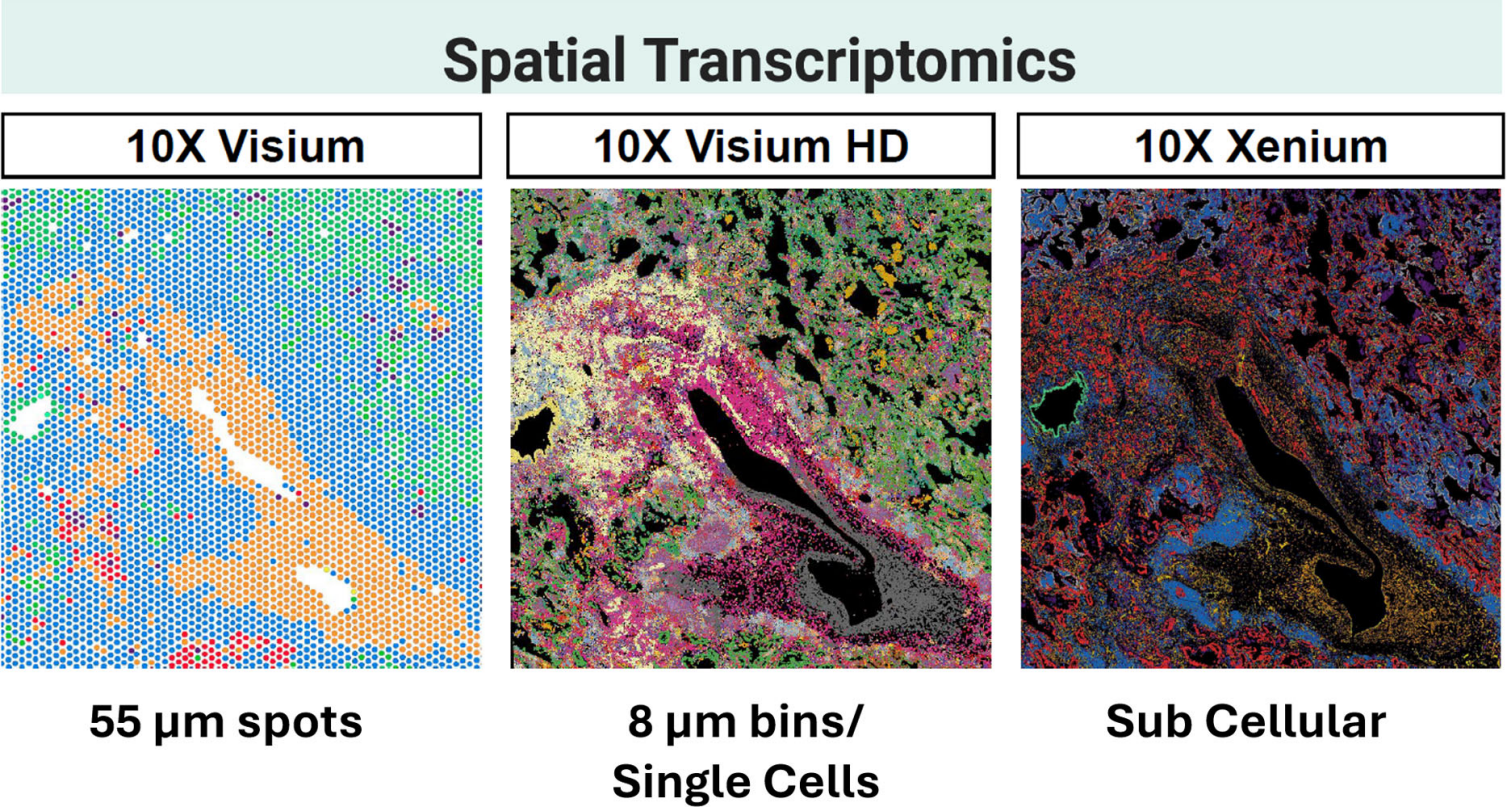
Application of spatial transcriptomics in non-small cell lung cancer (NSCLC) research. Spatially barcoded tissue slides enable the simultaneous capture of gene expression profiles and their spatial context within intact tissue sections. The 10X Genomics Visium platform employs a grid of capture spots, each typically encompassing transcripts from ~1–10 cells, whereas Visium HD provides substantially higher, near-subcellular resolution. The 10X Genomics Xenium platform enables single-cell–resolved spatial imaging of targeted gene panels, allowing the detection of up to ~5,000 genes across entire tissue sections. Shown are representative images of primary lung tumor tissue generated using 10X Visium (left), 10X Visium HD (center), and 10X Xenium (right), showing different achievable resolution. Captured spots are colored according to defined spatial clusters.

Many technologies exist today for assessing TIME and LIME using spatial proteomics (**Table 5**). For example, Imaging Mass Cytometry (IMC) uses metal-tagged antibodies, PhenoCycler/(previously CODEX) using oligos with fluorescent reporters, and CosMx using oligo-barcoded antibodies (**Figure 1**). Unlike spatial transcriptomics, spatial proteomics can resolve individual cell’s membrane boundary, better infer cell-cell contacts, and assess protein expression. These spatial technologies provide unprecedented depth and resolution for dissecting the TIME and LIME, enabling development of immune spatial biomarkers of prognostic value (**Table 4**). In LUAD, IMC study revealed that prognosis is shaped not merely by the overall abundance of immune infiltrates, but by their spatial localization, cell states, and organization into multicellular neighborhoods (201). High-grade patterns are often characterized by CD163+ Macrophage–Treg–enriched ecosystems, whereas low-grade disease more frequently exhibits tumor–T cell interaction patterns, and the survival advantage associated with B cell–enriched neighborhoods was abrogated when these regions were concomitantly enriched for Tregs (201).

Spatial proteomic and transcriptomic technologies reveal how these different cell types and cell states are arranged within lymph node architecture. Rather than being evenly distributed, immunosuppressive and immunostimulatory populations cluster into discrete micro-niches that can be mapped and quantified (298–300). In TSLNs, spatial analyzes highlight B cell follicular changes such as thinning or loss of mantle zones, altered GC organization, and ectopic plasma cell clusters (24, 272, 301, 302). Alterations in T cell zoning include Treg enrichment at the T–B border, and altered interactions between B cells and T helper cells (272, 303). Enriched spatial niches of myeloid populations where macrophages, DCs, and MDSCs, expressing checkpoint ligands and immunoregulatory cytokines co-localize with adaptive immune regions (24, 304, 305). Atypical stromal remodeling include changes in FRC networks that normally guide T cell movement, support B cell follicular architecture, and facilitate antigen transport (205, 207, 214, 298). By quantifying cell–cell distances, immune spatial niches, immune architecture, spatial platforms may transform qualitative impressions into quantitative metrics, such as B cell follicle integrity scores, Treg-mregDC niche scores, or myeloid suppressive niche signatures, which can be tested for association with recurrence or treatment response. When integrated with tumor data, spatial profiling of TSLNs enables mapping of coordinated or discordant patterns across compartments, for example whether TLS rich tumors tend to coexist with preserved or disrupted B cell follicular architecture in TSLNs, and how such LIME and TIME patterns relate to patient outcome.

## Summary and future directions

The transition from an anatomical TNM staging system to a biological framework for NSCLC necessitates a comprehensive understanding of the immune landscape. While current staging relies heavily on tumor size and the presence of metastasis, this approach fails to capture critical tumor-immune interactions. Accumulating evidence supports the TIME as a clinically meaningful biomarker space in NSCLC, while also underscoring that a tumor-centric view is inherently incomplete. Antitumor immunity is orchestrated across compartments in which the primary tumor reflects local immune engagement, whereas TSLN function as upstream immune “training hubs” that govern antigen presentation, T and B cell priming, affinity maturation, and durable memory formation. Multimodal single-cell and spatial platforms now enable high-resolution characterization of immune cell identity, cell state, clonal relationships, and spatial organization. These advances motivate an integrated TIME–LIME framework in which coordinated (or discordant) immune programs across tumor and TSLNs are quantified and linked to recurrence risk and therapeutic response, particularly in early-stage, node-negative disease where conventional staging reaches its prognostic limits.

Despite their transformative promise, current sequencing and spatial technologies retain important limitations that constrain interpretability and clinical translation. For single-cell profiling, tissue dissociation can introduce sampling bias by preferentially losing fragile populations and inducing artifactual activation programs. Transcriptomic measurements remain susceptible to dropout and limited sensitivity for low-abundance immune checkpoint transcripts, and RNA abundance does not always reflect protein-level activity due to post-transcriptional regulation. Spatial platforms preserve histologic context but impose trade-offs between gene sensitivity, field-of-view, and single-cell resolution. Furthermore, spatial transcriptomics may be limited by spot size or reduced transcript capture, whereas spatial proteomics is constrained by antibody availability/validation, channel capacity, imaging time, and segmentation errors that propagate into downstream neighborhood inference. Across both domains, variability in specimen handling (ischemia time, fixation, digestion conditions), chemistry versions, and analytic pipelines can produce batch effects that masquerade as biology, complicating cross-cohort comparisons and limiting reproducibility. Finally, cost, acquisition speed, and throughput remain major barriers, particularly for studies requiring paired tumor–TSLN sampling at scale, which is essential for robust integration of TIME and LIME signatures. Key strengths and limitations of different multiomics approaches are summarized in **Table 4**.

Accordingly, when selecting spatial technologies for TIME/LIME profiling, the optimal platform should be dictated by the biological question and the critical feature that must be preserved. Practical considerations include cost, acquisition speed, acquisition size (field-of-view and throughput), spatial resolution, antibody panel size, sample type compatibility (fresh frozen versus FFPE), and the unique strengths and weaknesses of each approach. In many contexts, the most informative strategy may not be the highest-plex assay, but rather the method that most faithfully resolves the key axis of interest, such as TLS maturation state, B cell follicle integrity and zoning, tumor–stroma barrier phenotypes, or stromal/vascular remodeling in TSLNs, while remaining scalable and reproducible across cohorts.

Looking forward, a major therapeutic opportunity is nodal-directed immunomodulation that treats TSLNs not merely as staging sites, but as actionable immune control centers. Because TSLNs are the dominant sites of priming and differentiation for tumor-reactive T and B cells, early remodeling toward immunosuppression may be a mechanistic driver of suboptimal antitumor immunity and subsequent recurrence. Future interventions may therefore aim to preserve or restore germinal center function, supporting productive antigen presentation, affinity maturation, and coordinated T–B collaboration. In parallel, reshaping stromal and vascular niches, such as FRC networks, HEVs, and lymphatic endothelial programs, could normalize lymphocyte trafficking, zoning, and APC–T cell interactions. At the cellular level, selective depletion or reprogramming of suppressive populations within TSLNs, including regulatory Tregs, MDSCs, mregDCs, and specific immunoregulatory macrophage states, represents a rational strategy to dismantle suppressive micro-niches. These approaches may be achieved using antibodies, small molecules, and immunotherapies tailored to nodal immune biology; in cases of severe, self-sustaining nodal dysfunction, more aggressive strategies that eliminate or neutralize dysregulated TSLNs may be considered to prevent node metastasis, though such approaches must balance therapeutic benefit against potential impairment of immune surveillance.

Equally important is the translation of high-dimensional discoveries into clinically feasible biomarkers. To impact patient management, the field will need simple, robust transcriptional or spatial biomarker panels that can be applied to routine surgical or biopsy specimens and that retain the most predictive information from TIME/LIME biology, such as B cell follicular integrity, progenitor-exhausted T cell niches, and suppressive myeloid–Treg neighborhoods. These assays should be coupled to inexpensive and automated assessment pipelines with standardized quality control, interpretable outputs, and harmonized scoring systems that facilitate prospective validation and clinical deployment. Ultimately, biomarker utility will depend on clear treatment associations, identifying who benefits from neoadjuvant versus adjuvant immunotherapy, who requires escalation or de-escalation, and which immune programs represent actionable vulnerabilities.

Finally, future work must explicitly incorporate the window of treatment and timing-dependent immunobiology. TIME and LIME states are dynamic across pre-treatment baseline, neoadjuvant exposure, post-resection immune remodeling, and recurrence, and distinct therapies may have different optimal windows depending on their reliance on intact priming in TSLNs versus intratumoral reinvigoration. Prospective pre-operative or sampling with longitudinal immune monitoring, integrated with clinical endpoints, will be essential to define when and where to intervene (tumor versus TSLN), which immune spatial features are most predictive at each stage, and how timing-guided therapeutic selection can improve outcomes for patients with early-stage NSCLC.
